# Supplementary material for: Tracking business opportunities for climate solutions using AI in regulated accounting reports
Source: Nat Commun. 2025 Nov 5;16:9769. doi: 10.1038/s41467-025-64723-1 (PMC12589618; doi:10.1038/s41467-025-64723-1)
Supplement: Supplementary file 1 — Supplementary Information [file 41467_2025_64723_MOESM1_ESM.pdf]

Supplementary Information

Supplementary Table 1: Variable Definition

Supplementary Table 2: Summary Statistics of Main Variables

Supplementary Table 3: Correlation of Alternative Constructions of Climate Solutions Measure

Supplementary Table 4: Validation of Climate Solutions Measure

Supplementary Table 5: Climate Solutions and Climate Risks

Supplementary Table 6: Climate Solutions and Revenue Growth

Supplementary Table 7: Climate Solutions and Political Affiliation of Firm Locations

Supplementary Table 8: Industry Climate Solutions Similarity and Stock Return Synchronicity

Supplementary Note 1: Climate Solutions GPT Model

Supplementary Note 2: Climate Solutions Labeling

Supplementary Table 1: Variable Definition

Variable	Description	Source
Climate Solutions Measure	The number of sentences classified as climate solutions divided by the total number of sentences in 10-K Item 1.	10-K
MSCI Green Revenue Percent	The percent of revenue that is related to low-carbon products.	MSCI
MSCI Green Patent Percent	The percent of patent value that is related to low-carbon products.	MSCI
R&D Expenses	Research and development expenses (xrd) divided by revenue (revt). This variable is coded as 0 if missing.	Compustat
Reported R&D (indicator)	An indicator for firm-year observations with non-missing R&D expenses.	Compustat
Knowledge Capital	Quantifies the stock of accumulated R&D investment by capitalizing R&D expenditures using industry-specific depreciation rates, divided by revenue (revt).	Ewens et al., 2024[1], Compustat
RDC	Research & Development Capitalization, capitalized value of R&D estimated based on its expected contribution to future revenues, divided by revenue (revt).	Iqbal et al., 2025[2], Compustat
Trade Secret	The number of trade secrets explicitly referenced in its 10-K filings, divided by revenue (revt).	Glaeser, 2018[3], Compustat
IRA Climate Solutions Measure	The number of sentences classified as climate solutions covered by IRA divided by the total number of sentences in 10-K Item 1.	10-K
Renewable Energy Topic	The number of sentences classified as climate solutions in the Renewable Energy Topic divided by the total number of sentences in 10-K Item 1.	10-K
Post IRA	Indicator for fiscal years 2021 and 2022, where annual reports are released in 2022 and 2023, after the IRA announcement.	10-K
Post RPS (weighted)	A weighted average of indicators for states after they pass renewable portfolio standards, where the weight is based on a firm's distribution of employees in each state.	10-K
Scope 1 GHG Absolute	Total scope 1 greenhouse gas emissions (di_319413).	TruCost
Scope 2 GHG Absolute	Total scope 2 greenhouse gas emissions (di_319414).	TruCost
Scope 3 GHG Upstream Absolute	Total upstream scope 3 greenhouse gas emissions (di_319415).	TruCost

Continued on following page

Supplementary Table 1, continued

Variable	Description	Source
Scope 3 GHG Downstream Absolute	Total downstream scope 3 greenhouse gas emissions (di_326737).	TruCost
Scope 1 GHG Intensity	Total scope 1 greenhouse gas emissions divided by revenue (di_319407).	TruCost
Scope 2 GHG Intensity	Total scope 2 greenhouse gas emissions divided by revenue (di_319408).	TruCost
Scope 3 GHG Upstream Intensity	Total upstream scope 3 greenhouse gas emissions divided by revenue (di_319409).	TruCost
Scope 3 GHG Downstream Intensity	Total downstream scope 3 greenhouse gas emissions divided by revenue (di_326738).	TruCost
MSCI Emissions Score	Emissions subscore from MSCI sustainability rating provider.	MSCI
Refinitiv Emissions Score	Emissions subscore from Refinitiv sustainability rating provider.	Refinitiv
Revenue	Annual revenue (revt), in millions USD, reported in annual reports.	Compustat
Age	Age of the firm since IPO, measured as years since the first appearance in Compustat.	Compustat
Revenue Growth	Percentage change in revenue, measured as (revenue - lagged revenue)/lagged revenue.	Compustat
Debt to Asset	Debt divided by asset.	Compustat
CAPEX to Asset	Capital Expenditure divided by asset.	Compustat
ROA	Net income divided by asset.	Compustat
3-Year Moving Average Revenue Growth	Average revenue growth from t=0 to t=2.	Compustat
High Abatement Potential	The number of sentences classified as climate solutions in in topics that belong to the High Abatement Potential group, divided by the total number of sentences in 10-K Item 1.	10-K
Low Abatement Potential	The number of sentences classified as climate solutions in in topics that belong to the High Abatement Potential group, divided by the total number of sentences in 10-K Item 1.	10-K
High Cost Per Abatement	The number of sentences classified as climate solutions in in topics that belong to the High Abatement Potential group, divided by the total number of sentences in 10-K Item 1.	10-K
Low Cost Per Abatement	The number of sentences classified as climate solutions in in topics that belong to the High Abatement Potential group, divided by the total number of sentences in 10-K Item 1.	10-K
>1% Climate Solutions Measure	An indicator for firm-year observations with Climate Solutions Measure greater than 1%.	10-K

Continued on following page

Supplementary Table 1, continued

Variable	Description	Source
>5% Climate Solutions Measure	An indicator for firm-year observations with Climate Solutions Measure greater than 5%.	10-K
SLVZ Climate Exposure	Climate exposure measure created using earnings conference call transcripts ($CCExposure$).	Sautner et al., 2023[4]
SLVZ Climate Opportunity	Climate exposure measure relating to opportunities created using earnings conference call transcripts ($CCExposure^{Opp}$).	Sautner et al., 2023[4]
Firms in Republican States	An indicator variable equal to 1 for firms located more in Republican voting states, defined as a firm's employee-weighted average Republican vote share across U.S. states (based on the 2020 presidential election) exceeding 50%.	InfoGroup
Synchronicity	Stock return synchronicity between two industry groups in a given month.	CRSP
Climate Solutions Similarity	Cosine similarity between the climate solution topic vectors of each industry group pair in each year.	10-K

This table provides the descriptions and sources of variables used in this paper.

Supplementary Table 2: Summary Statistics of Main Variables

	Count	Mean	Std. dev.	P25	P50	P75
Climate Solutions Measure	39710	2.410	6.271	0.000	0.000	1.492
MSCI Green Revenue Percent	1077	7.761	20.212	0.000	0.000	3.560
MSCI Green Patent Percent	682	11.297	20.725	0.000	2.950	10.375
R&D Expenses	39710	0.734	39.895	0.000	0.000	0.021
Reported R&D (indicator)	39710	0.510	0.500	0.000	1.000	1.000
Knowledge Capital	29158	1.216	51.945	0.000	0.000	0.058
RDC	27521	0.674	29.121	0.000	0.000	0.096
Trade Secret	37480	1.739	60.666	0.000	0.000	0.006
IRA Climate Solutions Measure	39710	2.187	6.032	0.000	0.000	1.176
Renewable Energy Topic	39710	0.793	3.325	0.000	0.000	0.000
Post IRA	39710	0.100	0.299	0.000	0.000	0.000
Post RPS (weighted)	22354	0.737	0.305	0.586	0.848	1.000
Scope 1 GHG Absolute (log)	13952	11.502	2.760	9.619	11.258	13.219
Scope 2 GHG Absolute (log)	13952	10.771	2.117	9.556	10.772	12.120
Scope 3 GHG Upstream Absolute (log)	13952	12.683	2.148	11.364	12.867	14.174
Scope 3 GHG Downstream Absolute (log)	6424	12.822	2.859	11.250	12.876	14.607
Scope 1 GHG Intensity (log)	13952	4.011	1.890	2.671	3.449	5.277
Scope 2 GHG Intensity (log)	13952	3.312	1.134	2.667	3.344	4.040
Scope 3 GHG Upstream Intensity (log)	13952	5.129	0.868	4.636	5.273	5.746
Scope 3 GHG Downstream Intensity (log)	6424	5.817	1.917	4.641	5.783	6.854
MSCI Emissions Score	9464	-0.000	1.000	-0.598	0.059	0.716
Refinitiv Emissions Score	11845	0.000	1.000	-1.050	-0.258	0.805
Revenue (log)	39710	5.766	2.671	4.178	6.248	7.689
Age (log)	39710	2.832	0.946	2.197	2.944	3.584
Revenue Growth	33200	0.128	0.507	-0.055	0.052	0.183
Debt to Asset	39479	0.392	0.800	0.084	0.273	0.434
CAPEX to Asset	39249	0.053	0.073	0.011	0.030	0.065
ROA	38803	-0.191	1.098	-0.040	0.028	0.069
3-Year Moving Average Revenue	37652	0.135	0.419	-0.019	0.057	0.174
High Abatement Potential	39710	1.305	4.659	0.000	0.000	0.501
Low Abatement Potential	39710	1.083	3.330	0.000	0.000	0.712
High Cost Per Abatement	39710	0.811	3.394	0.000	0.000	0.000
Low Cost Per Abatement	39710	1.502	4.415	0.000	0.000	0.926
>1% Climate Solutions Measure	39710	30.285	45.949	0.000	0.000	100.000
>5% Climate Solutions Measure	39710	12.977	33.605	0.000	0.000	0.000
SLVZ Climate Exposure	24887	-0.000	1.000	-0.433	-0.338	-0.066
SLVZ Climate Opportunity	24887	0.000	1.000	-0.333	-0.290	-0.151
Firms in Republican States	28109	0.621	0.485	0.000	1.000	1.000
Synchronicity	16848	-1.525	2.538	-1.918	-0.836	-0.067
Climate Solutions Similarity	16848	0.412	0.236	0.207	0.400	0.593

This table shows the summary statistics of the firm-year level variables used in the study.

Supplementary Table 3: Correlation of Alternative Constructions of Climate Solutions Measure

	(1)	(2)	(3)	(4)	(5)	(6)	(7)
(1) Climate Solutions Measure	1.000						
(2) CS Measure Top 50	0.926***	1.000					
(3) CS Measure Top 100	0.955***	0.955***	1.000				
(4) CS Measure Weighted 50	0.987***	0.965***	0.982***	1.000			
(5) CS Measure Weighted 100	0.992***	0.946***	0.982***	0.997***	1.000		
(6) CS Measure Rolling 50	0.919***	0.845***	0.890***	0.913***	0.922***	1.000	
(7) CS Measure Rolling 100	0.951***	0.877***	0.926***	0.946***	0.955***	0.984***	1.000

This table shows the correlation matrix of Climate Solutions Measure and six alternative construction methods.

Supplementary Table 4: Validation of Climate Solutions Measure

Panel A: Green Revenue and Green Patent

	MSCI Green Revenue Percent		MSCI Green Patent Percent	
	(1)	(2)	(3)	(4)
Climate Solutions Measure	1.512***	1.378***	1.728***	1.591***
	(9.88)	(8.91)	(12.48)	(10.25)
N	1077	1077	678	678
Adj. R-squared	0.280	0.432	0.355	0.395
Gind-year FE	No	Yes	No	Yes
Clusters	Firm	Firm	Firm	Firm

Panel B: Research and Development Expenses

	Dependent Variable = R&D Expenses					
	Full Sample		Low R&D Missing Industries		Exclude Missing R&D	
	(1)	(2)	(3)	(4)	(5)	(6)
Climate Solutions Measure	0.006***	0.004***	0.006***	0.003**	0.017***	0.011**
	(6.18)	(4.19)	(4.19)	(2.32)	(4.27)	(2.33)
Lagged Revenue (log)	-0.021***	-0.021***	-0.039***	-0.039***	-0.106***	-0.111***
	(-10.51)	(-10.69)	(-9.91)	(-9.95)	(-9.62)	(-9.80)
Age (log)	-0.007**	-0.014***	-0.019***	-0.027***	-0.041***	-0.064***
	(-2.33)	(-4.13)	(-3.08)	(-4.34)	(-2.60)	(-3.69)
Reported R&D (indicator)	0.110***	0.093***	0.141***	0.130***		
	(20.35)	(13.13)	(14.50)	(11.69)		
N	34588	34588	17411	17411	17248	17248
Adj. R-squared	0.146	0.185	0.168	0.206	0.132	0.141
Gind-year FE	No	Yes	No	Yes	No	Yes
Clusters	Firm	Firm	Firm	Firm	Firm	Firm

Panel C: Other Innovation Measures

	Knowledge Capital		RDC		Trade Secret	
	(1)	(2)	(3)	(4)	(5)	(6)
Climate Solutions Measure	0.018***	0.015***	0.011***	0.009***	0.020***	0.016***
	(5.83)	(4.45)	(5.88)	(4.57)	(4.98)	(3.39)
Lagged Revenue (log)	-0.074***	-0.072***	-0.052***	-0.047***	-0.143***	-0.148***
	(-11.31)	(-11.04)	(-11.06)	(-10.40)	(-12.04)	(-12.22)
Age (log)	0.009	-0.005	0.013*	-0.005	-0.000	-0.026*
	(1.02)	(-0.61)	(1.77)	(-0.68)	(-0.01)	(-1.67)
N	25046	25046	24327	24327	32919	32919
Adj. R-squared	0.136	0.191	0.114	0.243	0.136	0.147
Gind-year FE	No	Yes	No	Yes	No	Yes
Clusters	Firm	Firm	Firm	Firm	Firm	Firm

Continued on following page

Supplementary Table 4 (continued)

Panel D: Post IRA and Post RPS		
	(1)<br>IRA Climate<br>Solutions Measure	(2)<br>Renewable Energy<br>Topic
Post IRA X High IRA Industries	0.370***	
	(2.63)	
Post RPS (weighted) X High Renewable Industries		0.452**
		(2.48)
N	39293	21811
Adj. R-squared	0.908	0.878
Firm FE	Yes	Yes
Year FE	Yes	Yes
Clusters	Firm	Firm

This table shows validation of Climate Solutions Measure. Panel A shows the results of regressing MSCI green revenue and green patent on Climate Solutions Measure. Panel B shows the results of regressing R&D expenses on Climate Solutions Measure for different samples and specifications. Panel C shows the results of regressing knowledge capital, RDC and trade secret on Climate Solutions Measure. Panel D shows the results of regressing IRA climate solutions measure on post IRA and renewable energy topic on post RPS. The coefficients of interest on climate solutions measure are plotted in Fig. 2.

Supplementary Table 5: Climate Solutions and Climate Risks

Panel A: Absolute Greenhouse Gas Emissions

	Scope 1 GHG Absolute (log)		Scope 2 GHG Absolute (log)		Scope 3 GHG Upstream Absolute (log)		Scope 3 GHG Downstream Absolute (log)	
	(1)	(2)	(3)	(4)	(5)	(6)	(7)	(8)
Climate Solutions Measure	0.048***	-0.005	-0.030***	0.009*	0.023***	0.002	0.038***	-0.010
	(4.34)	(-0.90)	(-4.01)	(1.69)	(6.71)	(0.75)	(4.55)	(-1.06)
Lagged Revenue (log)	1.051***	0.899***	0.967***	1.003***	1.114***	1.026***	0.850***	0.873***
	(33.73)	(40.86)	(50.26)	(59.22)	(74.53)	(75.27)	(23.49)	(33.46)
Age (log)	0.331***	0.076*	-0.206***	-0.028	0.119***	0.005	-0.033	-0.092*
	(4.98)	(1.80)	(-4.82)	(-0.96)	(4.19)	(0.25)	(-0.50)	(-1.78)
N	13066	13066	13066	13066	13066	13066	6280	6280
Adj. R-squared	0.484	0.801	0.582	0.770	0.820	0.924	0.279	0.596
Gind-year FE	No	Yes	No	Yes	No	Yes	No	Yes
Clusters	Firm	Firm	Firm	Firm	Firm	Firm	Firm	Firm

Panel B: Intensity Greenhouse Gas Emissions

	Scope 1 GHG Intensity (log)		Scope 2 GHG Intensity (log)		Scope 3 GHG Upstream Intensity (log)		Scope 3 GHG Downstream Intensity (log)	
	(1)	(2)	(3)	(4)	(5)	(6)	(7)	(8)
Climate Solutions Measure	0.046***	-0.006	-0.022***	0.007	0.022***	0.001	0.043***	-0.006
	(4.18)	(-1.13)	(-3.98)	(1.54)	(6.23)	(0.34)	(5.96)	(-0.80)
Lagged Revenue (log)	0.062**	-0.082***	-0.023	0.008	0.111***	0.027**	-0.091***	-0.081***
	(2.14)	(-4.33)	(-1.50)	(0.56)	(8.11)	(2.47)	(-3.33)	(-4.39)
Age (log)	0.348***	0.100**	-0.136***	-0.001	0.152***	0.037*	0.051	0.003
	(5.44)	(2.50)	(-3.73)	(-0.04)	(5.54)	(1.93)	(0.90)	(0.09)
N	13066	13066	13066	13066	13066	13066	6280	6280
Adj. R-squared	0.044	0.666	0.022	0.475	0.103	0.678	0.022	0.620
Gind-year FE	No	Yes	No	Yes	No	Yes	No	Yes
Clusters	Firm	Firm	Firm	Firm	Firm	Firm	Firm	Firm

Continued on following page

Supplementary Table 5 (continued)

Panel C: Absolute Greenhouse Gas Emissions (Subsample with Board Sustainability Committee)

	Scope 1 GHG Absolute (log)		Scope 2 GHG Absolute (log)		Scope 3 GHG Upstream Absolute (log)		Scope 3 GHG Downstream Absolute (log)	
	(1)	(2)	(3)	(4)	(5)	(6)	(7)	(8)
Climate Solutions Measure	0.055***	0.002	-0.011	0.017**	0.026***	0.005	0.036***	-0.008
	(3.32)	(0.27)	(-1.15)	(2.46)	(5.10)	(1.18)	(2.81)	(-0.56)
Lagged Revenue (log)	1.058***	0.896***	0.957***	1.012***	1.150***	1.054***	0.790***	0.820***
	(22.91)	(26.10)	(35.62)	(40.96)	(52.65)	(51.05)	(14.33)	(19.93)
Age (log)	0.345***	0.065	-0.179***	-0.034	0.144***	-0.013	-0.023	-0.136*
	(3.80)	(1.11)	(-3.33)	(-0.91)	(3.82)	(-0.47)	(-0.26)	(-1.75)
N	8698	8698	8698	8698	8698	8698	4114	4114
Adj. R-squared	0.431	0.780	0.539	0.712	0.802	0.920	0.186	0.518
Gind-year FE	No	Yes	No	Yes	No	Yes	No	Yes
Clusters	Firm	Firm	Firm	Firm	Firm	Firm	Firm	Firm

Panel D: Intensity Greenhouse Gas Emissions (Subsample with Board Sustainability Committee)

	Scope 1 GHG Intensity (log)		Scope 2 GHG Intensity (log)		Scope 3 GHG Upstream Intensity (log)		Scope 3 GHG Downstream Intensity (log)	
	(1)	(2)	(3)	(4)	(5)	(6)	(7)	(8)
Climate Solutions Measure	0.050***	-0.001	-0.007	0.013**	0.023***	0.001	0.042***	-0.003
	(3.15)	(-0.19)	(-1.04)	(2.20)	(4.81)	(0.31)	(4.30)	(-0.30)
Lagged Revenue (log)	0.063	-0.089***	-0.036*	0.017	0.140***	0.049***	-0.124***	-0.110***
	(1.47)	(-2.94)	(-1.66)	(0.81)	(7.10)	(2.91)	(-3.10)	(-3.81)
Age (log)	0.354***	0.080	-0.120**	-0.010	0.173***	0.011	0.061	-0.040
	(4.06)	(1.44)	(-2.53)	(-0.28)	(4.70)	(0.46)	(0.82)	(-0.74)
N	8698	8698	8698	8698	8698	8698	4114	4114
Adj. R-squared	0.043	0.668	0.013	0.409	0.127	0.707	0.020	0.589
Gind-year FE	No	Yes	No	Yes	No	Yes	No	Yes
Clusters	Firm	Firm	Firm	Firm	Firm	Firm	Firm	Firm

Continued on following page

Supplementary Table 5 (continued)

Panel E: ESG Rating Emissions Score

	MSCI Emissions Score		Refinitiv Emissions Score	
	(1)	(2)	(3)	(4)
Climate Solutions Measure	0.019***	0.017***	0.020***	0.016***
	(4.33)	(3.35)	(5.41)	(3.81)
Lagged Revenue (log)	0.165***	0.233***	0.279***	0.345***
	(10.34)	(17.79)	(23.60)	(26.11)
Age (log)	0.129***	0.073***	0.157***	0.142***
	(4.10)	(3.17)	(6.18)	(5.57)
N	9269	9269	11209	11209
Adj. R-squared	0.099	0.576	0.310	0.439
Gind-year FE	No	Yes	No	Yes
Clusters	Firm	Firm	Firm	Firm

This table shows the relation between climate solutions and climate risks. Panel A (B) shows the results of regressing scopes 1, 2, 3 upstream, and 3 downstream absolute (intensity) greenhouse gas emissions on Climate Solutions Measure. Panels C and D repeat the specifications in panels A and B, limiting to a subsample with a sustainability committee on their board, where environmental data is likely more reliable. Panel E shows the results of regressing MSCI and Refinitiv emissions scores on Climate Solutions Measure. All regressions control for log revenue in period t-1 and firm age, and show specifications with and without industry-year fixed effects. The coefficients of interest on Climate Solutions Measure in Panels A and B are plotted in Fig. 3.

Supplementary Table 6: Climate Solutions and Revenue Growth

Panel A: Baseline Results and Cross Sectional by Patents

	Dependent Variable = Revenue Growth					
	(1)	(2)	(3)	(4)	(5)	(6)
Climate Solutions Measure	0.004***	0.003***	0.005***	0.005***	0.002*	0.002
	(4.51)	(3.82)	(5.00)	(4.60)	(1.70)	(1.02)
Lagged Revenue (log)	-0.037***	-0.036***	-0.029***	-0.029***	-0.045***	-0.044***
	(-16.73)	(-15.23)	(-10.43)	(-9.85)	(-12.78)	(-11.61)
Age (log)	-0.070***	-0.072***	-0.057***	-0.062***	-0.077***	-0.081***
	(-15.47)	(-15.00)	(-9.18)	(-9.71)	(-11.77)	(-11.35)
Debt to Asset	-0.037***	-0.047***	-0.044***	-0.049***	-0.040**	-0.046**
	(-3.21)	(-3.96)	(-3.17)	(-3.44)	(-2.11)	(-2.39)
CAPEX to Asset	0.990***	0.935***	1.100***	0.926***	0.912***	0.912***
	(11.97)	(9.41)	(6.63)	(5.15)	(9.43)	(7.76)
ROA	0.012	-0.001	-0.008	-0.016	0.028	0.016
	(1.08)	(-0.09)	(-0.58)	(-1.31)	(1.51)	(0.87)
N	32861	32861	16537	16537	16324	16324
Adj. R-squared	0.081	0.149	0.069	0.129	0.093	0.164
Gind-year FE	No	Yes	No	Yes	No	Yes
Subsample			High Patent		Low Patent	
Clusters	Firm	Firm	Firm	Firm	Firm	Firm

Panel B: Topic characteristic

	Dependent Variable = Revenue Growth							
	(1)	(2)	(3)	(4)	(5)	(6)	(7)	(8)
High Cost per Abatement	0.018***	0.013**						
	(2.97)	(1.97)						
Low Cost per Abatement			0.019***	0.017***				
			(4.24)	(3.49)				
High Abatement Potential					0.025***	0.023***		
					(4.91)	(4.47)		
Low Abatement Potential							0.008*	0.006
							(1.66)	(1.14)
Lagged Revenue (log)	-0.037***	-0.036***	-0.038***	-0.037***	-0.037***	-0.036***	-0.038***	-0.037***
	(-16.65)	(-15.26)	(-16.85)	(-15.38)	(-16.67)	(-15.25)	(-16.78)	(-15.40)
Age (log)	-0.071***	-0.073***	-0.071***	-0.072***	-0.071***	-0.072***	-0.071***	-0.074***
	(-15.61)	(-15.27)	(-15.48)	(-15.15)	(-15.57)	(-15.10)	(-15.57)	(-15.34)
Debt to Asset	-0.037***	-0.047***	-0.038***	-0.047***	-0.036***	-0.046***	-0.038***	-0.047***
	(-3.24)	(-3.97)	(-3.29)	(-4.00)	(-3.16)	(-3.94)	(-3.34)	(-4.01)
CAPEX to Asset	0.993***	0.930***	0.991***	0.938***	0.993***	0.923***	0.994***	0.938***
	(11.98)	(9.33)	(11.99)	(9.47)	(12.00)	(9.30)	(12.01)	(9.46)
ROA	0.011	-0.002	0.010	-0.002	0.012	-0.001	0.009	-0.002
	(0.98)	(-0.15)	(0.94)	(-0.16)	(1.11)	(-0.06)	(0.82)	(-0.21)
N	32861	32861	32861	32861	32861	32861	32861	32861
Adj. R-squared	0.080	0.148	0.080	0.148	0.081	0.149	0.079	0.148
Gind-year FE	No	Yes	No	Yes	No	Yes	No	Yes
Clusters	Firm	Firm	Firm	Firm	Firm	Firm	Firm	Firm

Continued on following page

Supplementary Table 6 (continued)

Panel C: Robustness tests using alternative revenue growth specifications

	1-Year Forward Revenue Growth		3-Year MA Revenue Growth		
	(1)	(2)	(3)	(4)	(5)
Climate Solutions Measure	0.003***	0.003***	0.004***	0.003***	0.004*
	(3.65)	(2.92)	(4.22)	(3.27)	(1.82)
Lagged Revenue (log)	-0.029***	-0.028***	-0.031***	-0.031***	-0.245***
	(-13.24)	(-12.07)	(-12.91)	(-12.36)	(-22.73)
Age (log)	-0.041***	-0.043***	-0.044***	-0.046***	-0.074***
	(-9.86)	(-9.87)	(-9.95)	(-9.85)	(-3.24)
Debt to Asset	-0.040***	-0.052***	-0.033***	-0.043***	-0.049***
	(-3.28)	(-3.96)	(-2.85)	(-3.53)	(-3.14)
CAPEX to Asset	0.442***	0.548***	0.717***	0.746***	0.340***
	(6.06)	(6.11)	(9.90)	(8.86)	(3.95)
ROA	-0.039***	-0.045***	-0.009	-0.016	0.012
	(-3.28)	(-3.74)	(-0.81)	(-1.42)	(0.70)
N	28801	28801	32680	32678	32680
Adj. R-squared	0.052	0.138	0.094	0.152	0.541
Gind-year FE	No	Yes	No	Yes	No
Year FE	No	No	No	No	Yes
Firm FE	No	No	No	No	Yes
Clusters	Firm	Firm	Firm	Firm	Firm

Panel D: Entropy balanced samples

	Dependent Variable = Revenue Growth			
	(1)	(2)	(3)	(4)
>1% Climate Solutions Measure	0.029***	0.030***		
	(3.96)	(4.52)		
>5% Climate Solutions Measure			0.033***	0.036***
			(2.78)	(3.20)
Lagged Revenue (log)	-0.037***	-0.030***	-0.037***	-0.037***
	(-15.50)	(-13.86)	(-15.38)	(-11.64)
Age (log)	-0.074***	-0.058***	-0.073***	-0.047***
	(-15.41)	(-14.08)	(-15.34)	(-8.36)
Debt to Asset	-0.047***	-0.040***	-0.047***	-0.054***
	(-3.96)	(-3.02)	(-3.98)	(-3.77)
CAPEX to Asset	0.940***	0.739***	0.935***	0.961***
	(9.45)	(8.73)	(9.41)	(7.89)
ROA	-0.001	0.001	-0.002	-0.007
	(-0.13)	(0.07)	(-0.15)	(-0.52)
N	32861	32861	32861	32861
Adj. R-squared	0.148	0.143	0.148	0.141
Entropy Balanced	No	Yes	No	Yes
Gind-year FE	Yes	Yes	Yes	Yes
Clusters	Firm	Firm	Firm	Firm

Continued on following page

Supplementary Table 6 (continued)

Panel E: Controlling for SLVZ Climate Measures

	Dependent Variable = Revenue Growth			
	(1)	(2)	(3)	(4)
Climate Solutions Measure	0.005***	0.004***	0.005***	0.004***
	(4.41)	(4.12)	(4.72)	(4.00)
SLVZ Climate Exposure	0.002	-0.006		
	(0.37)	(-1.08)		
SLVZ Climate Opportunity			-0.001	-0.005
			(-0.20)	(-1.09)
Lagged Revenue (log)	-0.044***	-0.040***	-0.044***	-0.040***
	(-12.70)	(-11.70)	(-12.70)	(-11.68)
Age (log)	-0.053***	-0.055***	-0.052***	-0.055***
	(-11.57)	(-11.66)	(-11.58)	(-11.66)
Debt to Asset	0.028	-0.007	0.029	-0.007
	(1.46)	(-0.33)	(1.46)	(-0.32)
CAPEX to Asset	0.851***	0.640***	0.852***	0.642***
	(10.35)	(6.22)	(10.38)	(6.23)
ROA	0.237***	0.147***	0.237***	0.147***
	(3.60)	(2.59)	(3.60)	(2.59)
N	22550	22550	22550	22550
Adj. R-squared	0.096	0.209	0.096	0.209
Gind FE	No	Yes	No	Yes
Clusters	Firm	Firm	Firm	Firm

This table shows the results of regressing revenue growth on Climate Solutions Measure. Panel A shows the baseline results and cross sectional analysis separating industries with high and low patents. Panel B shows the results replacing Climate Solutions Measures with measures based on topics with high or low abatement potential and cost per abatement. These four topic measures are standardized for ease of comparison. Panel C provide robustness tests showing results using alternative specifications of revenue growth using the one-year forward revenue growth in columns 1 and 2, and the moving average revenue growth from periods t=0 to t=2. Panel D shows results of using entropy balanced samples based on whether or not a firm has more than 1% or 5% Climate Solutions Measure. Panel E shows results controlling for SLVZ Climate Exposure and Opportunity measures. All regressions control for log revenue in period t-1, firm age, debt to asset, CAPEX to asset, and ROA. We show specifications with and without industry-year fixed effects. Panel C column 5 shows the specification with firm fixed effects and year fixed effects. The coefficients of interest on Climate Solutions Measure in Panels A and B are plotted in Fig. 7.

Supplementary Table 7: Climate Solutions and Political Affiliation of Firm Locations

Panel A: Political Affiliation based on 2020 Presidential Election

	Climate Solutions Measure		High Cost Per Abatement		Low Cost Per Abatement		High Abatement Potential		Low Abatement Potential	
	(1)	(2)	(3)	(4)	(5)	(6)	(7)	(8)	(9)	(10)
Firms in Republican States	-0.246	-0.529**	-0.086**	-0.140***	0.004	-0.028	-0.044	-0.028	-0.012	-0.119**
	(-0.93)	(-1.99)	(-2.01)	(-2.94)	(0.09)	(-0.64)	(-1.06)	(-0.67)	(-0.28)	(-2.42)
Lagged Revenue (log)	-0.200***	-0.221***	-0.034***	-0.038***	-0.013	-0.018*	-0.030***	-0.029***	-0.018**	-0.025***
	(-3.41)	(-4.04)	(-3.53)	(-4.68)	(-1.45)	(-1.78)	(-3.06)	(-3.19)	(-2.01)	(-2.72)
Age (log)	-0.637***	-0.792***	-0.070***	-0.095***	-0.094***	-0.110***	-0.076***	-0.109***	-0.085***	-0.085***
	(-4.34)	(-5.70)	(-3.31)	(-4.55)	(-3.70)	(-4.54)	(-3.23)	(-4.88)	(-3.42)	(-3.55)
N	25079	25079	25079	25079	25079	25079	25079	25079	25079	25079
Adj. R-squared	0.021	0.269	0.019	0.195	0.010	0.159	0.016	0.269	0.010	0.102
Gind-year FE	No	Yes	No	Yes	No	Yes	No	Yes	No	Yes
Clusters	Firm	Firm	Firm	Firm	Firm	Firm	Firm	Firm	Firm	Firm

Panel B: Political Affiliation based on 2016 Presidential Election

	Climate Solutions Measure		High Cost Per Abatement		Low Cost Per Abatement		High Abatement Potential		Low Abatement Potential	
	(1)	(2)	(3)	(4)	(5)	(6)	(7)	(8)	(9)	(10)
Firms in Republican States	-0.333	-0.521*	-0.109**	-0.149***	-0.000	-0.023	-0.057	-0.028	-0.019	-0.116**
	(-1.27)	(-1.95)	(-2.56)	(-3.23)	(-0.01)	(-0.53)	(-1.39)	(-0.66)	(-0.45)	(-2.43)
Lagged Revenue (log)	-0.197***	-0.218***	-0.033***	-0.037***	-0.013	-0.018*	-0.029***	-0.029***	-0.017**	-0.024***
	(-3.37)	(-4.00)	(-3.48)	(-4.63)	(-1.45)	(-1.78)	(-3.03)	(-3.18)	(-1.99)	(-2.67)
Age (log)	-0.636***	-0.791***	-0.070***	-0.095***	-0.094***	-0.110***	-0.075***	-0.109***	-0.085***	-0.084***
	(-4.33)	(-5.70)	(-3.31)	(-4.56)	(-3.70)	(-4.54)	(-3.22)	(-4.89)	(-3.42)	(-3.54)
N	25079	25079	25079	25079	25079	25079	25079	25079	25079	25079
Adj. R-squared	0.022	0.269	0.020	0.196	0.010	0.159	0.016	0.269	0.010	0.102
Gind-year FE	No	Yes	No	Yes	No	Yes	No	Yes	No	Yes
Clusters	Firm	Firm	Firm	Firm	Firm	Firm	Firm	Firm	Firm	Firm

This table shows the results of regressing Climate Solutions Measure on an indicator for firms located more in Republican voting states. All regressions control for log revenue in period t-1 and firm age, and show specifications with and without industry-year fixed effects. Panel A shows results where the political affiliation of each state is based on 2020 presidential election outcomes. Panel B shows results where the political affiliation of each state is based on 2016 presidential election outcomes. In both panels, the dependent variables in Columns 3 to 10 are standardized for ease of comparison.

Supplementary Table 8: Industry Climate Solutions Similarity and Stock Return Synchronicity

	(1)<br>Synchronicity	(2)<br>Synchronicity
Climate Solutions Similarity	1.786***	1.654***
	(3.47)	(3.06)
N	16848	16848
Adj. R-squared	0.028	0.229
Year-month FE	No	Yes
Clusters	Industry group-pair	Industry group-pair

This table shows the results of regressing monthly stock return synchronicity on Climate Solutions Similarity for pairs of GICS industry groups. We show specifications with and without year-month fixed effects. The coefficients of interest on Climate Solutions Similarity are plotted in Fig. 10b.

Supplementary Note 1: Climate Solutions GPT Model

This note details the methodology employed in developing the climate solutions GPT model and its application in identifying climate solutions topics. A high-level overview of these methodological steps is illustrated in Supplementary Fig. 1. In order to facilitate a comprehensive understanding of our methodological framework, we have organized the document into sections that correspond with each phase of our process. First, we discuss the dataset and its preparation. Second, we discuss our methodology for developing the binary classification model to identify climate solutions. Third, we detail procedures undertaken to extract topics associated with climate solutions.

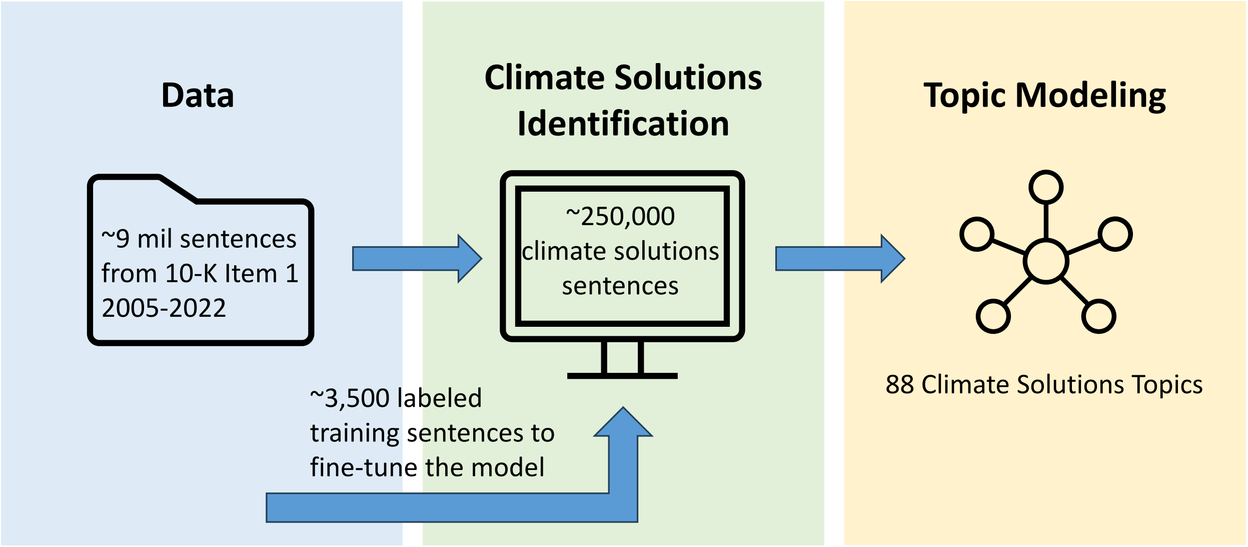

Supplementary Fig. 1: Overview of methodological steps

Data and Sample

Our primary data source is the SEC's EDGAR database, offering public access to 10K filings. A 10K filing is an annual report filed by publicly traded companies in the United States. As a regulatory document, filed with the Securities and Exchange Commission (SEC), companies are required to present factual information, which makes the report more reliable than other sources like sustainability reports and earnings conference calls. The report contains detailed information about a company's overall financial health, business practices, and strategy. Climate solutions are related to the product offering of companies therefore, for our analysis, we specifically targeted the business descriptions found in Part I, Item 1 (Business) of these filings.

Our sample starts with the universe of firms that report SEC 10-K filing in the EDGAR database from fiscal year 2005 to 2022. Our sample period begins in 2005 when the structure of 10-K is more stable. Starting 2005, the Securities and Exchange Commission (SEC) requires firms to disclose the most significant risks in Item 1A (Securities Offering Reform, Item 503(c) of Regulation S-K).

To ensure consistent firm identifiers over time, we use the WRDS-CIK linking tables to map the CIK in 10-K filings to GVKEY in Compustat[5]. This linking table allows us to match firms in Compustat to its historical CIK that could be different from the latest CIK due to firm name and structure changes (e.g., merger and acquisition, spin-offs, and bankruptcies). For example,

General Motors filed for bankruptcy in 2009 and received a new CIK following that year. We are able to assign both CIK before and after the bankruptcy to the same GVKEY. We keep firm-year observations that are matched to Compustat as the majority of the firms not matched are funds, which we exclude together with financial institutions since we focus on climate solution products and services, but not the financing of them. Supplementary Table 9 shows the sample composition, where 37% of observations are excluded as a result of this requirement. We then use the Extractor API (Python) from the SEC API to retrieve the raw text of the Item 1 business description section of the 10-K filings. This process results in the loss of around 1% of observations where the API was not able to identify Item 1 or that the identified Item 1 contains fewer than 100 words.

We focus on industries that are pivotal to climate solutions, where our LLM is likely more accurate in identifying climate solutions. Based on reviewing Project Drawdown, we keep 13 (out of 25) GICS industry groups that are central to climate solutions: Energy, Materials, Capital Goods, Transportation, Automobiles & Components, Consumer Durables & Apparel, Food Beverage & Tobacco, Household & Personal Products, Technology Hardware & Equipment, Semiconductors & Semiconductor Equipment, Utilities, Equity Real Estate Investment Trusts (REITs), Real Estate Management & Development. This restriction reduces the sample by 35%. This process results in a final sample of 39,710 observations for 4,483 firms for fiscal years 2005 to 2022.

Supplementary Table 9: Sample Composition

Sample Composition		
	Sample Size	Ratio
Total 10K from edgar 2005-2022	146,718	
Firms not matched to Compustat	(53,894)	37%
Firms unable to extract item 1	(1,575)	1%
Firms not in relevant industries	(51,539)	35%
Final sample	39,710	

This table shows the sample composition.

Climate Solutions Identification

The basis of our metric is a sentence-level binary classifier, designed to detect the presence of climate solutions within the text. This model was specifically developed for sentence-level classification (climate solution or not) due to two primary considerations. First, a sentence, as the fundamental unit of text, presents a clear and concise element for labelers to assess with high accuracy. Second, this method ensures the precise extraction and identification of text segments specifically relevant to climate solutions.

Defining Climate Solutions

We define climate solutions as products and services that develop or deploy new technologies in a transition to a low-carbon economy. We identify climate solution technologies based on guidance from Project Drawdown. Project Drawdown contains a list of technologies that can reduce greenhouse gases in the atmosphere, and are compiled by a network of scientists and researchers.

While Project Drawdown provides guidance on what decarbonization technology is considered a climate solution, when we label sentences, we need to decide for which firms the climate solution is a relevant product or service. Consider the following example with three companies involved in the climate solution technology of sustainable aviation fuel (SAF): an energy producer provides SAF to airlines to reduce its emissions and the airline sells flight tickets with lower carbon footprint

to a consulting firm. We consider SAF a relevant climate solution for the energy producer since it is the developer of the technology. We also consider SAF a relevant climate solution for the airline since it deploys the technology. However, we do not consider SAF a relevant climate solution for the consulting firm since it engages in business as usual and neither develops nor deploys the climate solution technology.

Creating the training dataset

In the full dataset of almost nine million sentences from 10-K Item 1, only some of them pertain to climate solutions. Therefore, it is crucial to focus on the most representative sentences for efficient training of the model. We select sentences as our training dataset in two steps. In the first step, we select a sample of 100 sentences from each of the 13 industry groups based on sentences most confusing to the model using a one-shot BART model from Setfit. In the one-shot BART model, we predict whether a sentence is a climate solution sentence based on its alignment with Project Drawdown's Solutions Library. By using a BART model instead of randomly selecting sentences, we also ensure a better balance between positive and negative sentences. These chosen sentences go through a labeling process, which we describe in more detail in Supplementary Note 2.

In the second step, we conduct an iterative process to add sentences to the training set through an active learning approach. Active learning is a machine learning technique where the model identifies and selects specific data points for which it requires additional information (labels or annotations) to improve its performance. The technique often involves selecting data points where the model is uncertain. Thus, we identify common types of sentences that our model struggles to interpret or predicts as climate solutions (e.g., sentences considering climate regulations), and we include additional sentences on these confusing areas to further enhance the model. The objective of active learning is to select the data points from which the model learns better, aiming to improve learning efficiency and performance with less labeled data. This approach is particularly useful in scenarios where labeling data is expensive or time-consuming. By focusing on instances where the model's prediction is uncertain, active learning seeks to minimize the amount of required training data, thereby reducing costs and improving the model's accuracy and generalization capabilities.

We use a pre-trained ClimateBERT machine learning model as the base model for the active learning processes[6]. A BERT model has the ability to capture rich contextual information, thus identifying and understanding ambiguous or uncertain cases. This capability enhances the effectiveness of the active learning process by ensuring that the most informative and challenging examples are selected for labeling. The ClimateBERT model's relatively compact size (in its number of weights/parameters) offers the advantage of requiring minimal computational power, enabling comparably quick fine-tuning. To mitigate the drawback of a smaller size model and less context encoded in its weights, the authors of ClimateBERT pre-trained it further on over 2 million paragraphs of climate-related texts to better respond to the domain-specific queries. Like any other binary classification model, ClimateBERT returns a logit, which can be transformed back to probabilities using a logistic function. Based on this output, we conduct the following iterative process:

1. Fine-tuned the model with the data.

2. Choose a decision boundary, that guarantees the highest F1 score.

3. Carefully examine the sentences whose predictions are close to the decision boundary.

4. Use these to guide the addition of new sentences into the dataset.

We underwent 8 rounds of active learning and generating training sets, as listed below. For each round, we identify the type of sentences causing confusion to the model and add around 200

sentences to the training set.

1. Sentences that contain "battery" or "electric" but are not related to climate solutions, such as those containing electric toothbrushes.

2. Sentences that describe climate policies or regulations faced by the firm, which does not mean the firm has products or services on climate solutions.

3. Sentences associated with buying carbon credits (e.g., renewable energy credits), but not the creation of carbon credits.

4. Sentences in the building/construction industry that likely needed more examples to properly inform the classifier's decision boundary, specifically when it relates to green buildings and LEED certifications.

5. Sentences containing ethanol, as the model initially does not consider most mentions of ethanol production as climate solution.

6. Sentences where the prefix 'bio' is present, where the model initially classifies as climate solutions but many are not, such as BiOmega-3.

7. Sentences containing generic agricultural products are sometimes misclassified as climate solutions, whereas sentences related to nutrient management and plant-based protein are climate solutions.

8. Sentences containing supporting products to other climate solutions are sometimes not classified as climate solutions. For example, products that enable existing cars to transition to a less carbon-intensive fuel.

This process results in a final training set of 3,508 sentences. The training set statistics are presented in Supplementary Table 10. The size of our dataset is benchmarked to Stammbach et al., 2023, where they annotated 3000 sentences to fine-tune transformer models for climate claim detection[7]. Additionally, we evaluate the sufficiency of our training set size by examining how model performance changes as we increase the size of the training dataset. Specifically, we keep a held-out dataset using 20% of the training set, and examine the model performance on this held-out set when we train a GPT-3.5-turbo-1106 model using 0%, 20%, 40%, 60%, and 80% of the training set. Supplementary Fig. 2 shows the largest increase in model performance when the model is fine-tuned with 20% of the training set, compared to the non-fine-tuned model when 0% training data is provided. This increase reflects the value of fine-tuning the GPT model for the specific task of identifying climate solutions sentences. As the proportion of training set increases from 20% to 80%, we do not observe large improvements in model performance, which provides comfort that our training set is sufficient and that we do not anticipate large improvements in model performance if we were to annotate additional sentences.

Supplementary Table 10: Composition of the training data

Industry Group	Count in Training Set	Number of Positives	% of Positives	Count Overall	% of the Training Set	% of Overall Set
Automobiles and Components	291	168	0.577	180,942	8.640	1.984
Capital Goods	405	139	0.343	1,238,834	12.025	13.588
Consumer Durables and Apparel	178	45	0.253	494,283	5.285	5.421
Energy	181	74	0.409	1,769,351	5.374	19.406
Equity Real Estate Investment Trusts	188	57	0.303	492,869	5.582	5.406
Food, Beverage and Tobacco	451	163	0.361	412,178	13.391	4.521
Household and Personal Products	146	18	0.123	220,420	4.335	2.418
Materials	301	71	0.236	896,885	8.937	9.837
Real Estate Management and Development	134	40	0.299	492,869	3.979	5.406
Semiconductors and Semiconductor Equipment	178	69	0.388	460,569	5.285	5.052
Technology Hardware and Equipment	158	33	0.209	917,277	4.691	10.061
Transportation	184	46	0.250	313,834	5.463	3.442
Utilities	573	331	0.578	1,227,056	17.013	13.458

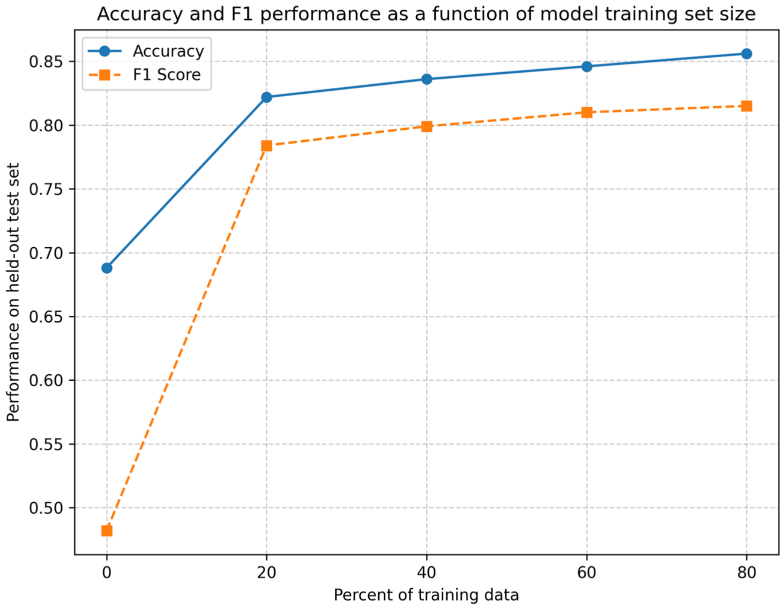

Supplementary Fig. 2: Model Performance relative to Training Size

Training Methodology and Model Selection

We use the labeled training set to fine-tune a GPT-3.5-turbo-1106 specialized at labeling climate solutions sentences. Fine-tuning is the process of further training a pre-trained GPT model on a specific data and involves adjusting the model's weights to better capture the language and concepts related to climate solutions. GPT algorithm is based on a neural network architecture that depends on weights, which are the parameters that are learned during training. The weights determine the strength of connections between neurons in different layers of the model. Adjusting these weights changes the way the model processes input data and generates output. Fine-tuning adjusts the model's weights so it can better understand and generate climate-specific terms and phrases,

such as “renewable energy,” “plant-based protein,” and “cogeneration.” Through this process, the model learns the contextual usage of these terms within climate-related discussions, improving its ability to generate relevant and coherent text specific to climate solutions. The fine-tuning hyperparameters for our GPT-based model are based on recommended defaults, with epochs set to 3, batch size to 7, and a learning rate multiplier of 2.

We employ five-fold cross-validation to assess our model, optimizing the use of our labeled dataset. This method ensures comprehensive evaluation by partitioning the dataset into five parts (folds) and running five iterations. In each iteration, four folds (80%) are used to fine-tune the GPT-3.5-turbo-1106 model, and the remaining fold (20%) is used as a validation set. We report model performance as the average across the five iterations. When presenting results by industry, we present average performance metrics using only the iteration in which each sentence appears in the validation set.

The model demonstrates an average accuracy of 84.09% across five folds, with a standard deviation of 1.93% between folds, indicating consistency in performance across different subsets. Moreover, we report an average F1 score of 79.50% across five folds with a standard deviation of 2.32% between the folds. The F1 score, being the harmonic mean of precision (the percentage of predicted positives that are truly positive) and recall (the percentage of true positives that are predicted as positives), provides a balanced measure of the model’s accuracy, particularly valuable in the context of binary classification. It is especially pertinent for evaluating performance in imbalanced datasets, where traditional accuracy metrics may not fully capture the effectiveness of the model in distinguishing between the binary classification.

We display the GPT prompt below and the detailed model performance by industry in Supplementary Table 11.

Listing 1: GPT finetuning prompt

```
system_message =  You  are a chatbot with expertise in
   environmental regulations and climate change mitigation
   strategies. Your function is to meticulously analyze sections
   of regulatory documents, 10k filings, to identify the presence
    of proposed climate solutions. Based on the guidelines,
   assess whether the company is implementing specific
   technologies or practices contributing to a low-carbon economy
   . Look for whether there is a clear indication of the
   companys investment or future investment in climate
   solutions or the sentence implies a reduction in carbon
   emissions through the companys products or services.
   Generic, vague, or general statements about climate change
   should classified as no.
```

Supplementary Table 11: Model Evaluation by Industry

	GPT 3.5 FT		ClimateBERT FT		EnvironmentalBERT FT		RoBERTa FT		DistilRoBERTa FT		DeBERTa FT	
**Industry Group**	**F1 Score**	**Accuracy**	**F1 Score**	**Accuracy**	**F1 Score**	**Accuracy**	**F1 Score**	**Accuracy**	**F1 Score**	**Accuracy**	**F1 Score**	**Accuracy**
Automobiles and Components	0.85	0.82	0.84	0.81	0.82	0.80	0.86	0.82	0.80	0.78	0.82	0.79
Utilities	0.84	0.82	0.84	0.80	0.85	0.82	0.85	0.82	0.84	0.81	0.82	0.79
Real Estate Management and Development	0.84	0.90	0.71	0.81	0.87	0.93	0.79	0.88	0.74	0.85	0.72	0.84
Energy	0.83	0.87	0.83	0.86	0.82	0.85	0.82	0.85	0.79	0.83	0.82	0.86
Food, Beverage and Tobacco	0.79	0.84	0.80	0.84	0.79	0.84	0.79	0.83	0.79	0.84	0.80	0.84
Capital Goods	0.79	0.85	0.77	0.83	0.81	0.86	0.75	0.82	0.75	0.82	0.76	0.82
Consumer Durables and Apparel	0.78	0.88	0.73	0.85	0.80	0.89	0.75	0.85	0.67	0.81	0.70	0.84
Technology Hardware and Equipment	0.73	0.89	0.65	0.85	0.58	0.83	0.64	0.82	0.61	0.83	0.54	0.82
Semiconductors and Semiconductor Equipment	0.72	0.76	0.72	0.76	0.72	0.76	0.70	0.72	0.70	0.75	0.72	0.76
Transportation	0.72	0.85	0.67	0.82	0.64	0.82	0.66	0.80	0.59	0.79	0.65	0.82
Materials	0.70	0.85	0.65	0.82	0.65	0.81	0.65	0.81	0.62	0.80	0.65	0.81
Equity Real Estate Investment Trusts	0.69	0.80	0.63	0.74	0.68	0.79	0.60	0.74	0.59	0.73	0.66	0.79
Household and Personal Products	0.64	0.89	0.53	0.83	0.55	0.84	0.50	0.82	0.53	0.83	0.59	0.86
**Overall**	**0.80**	**0.84**	**0.78**	**0.82**	**0.79**	**0.83**	**0.78**	**0.82**	**0.76**	**0.81**	**0.76**	**0.82**

In the process of selecting an appropriate LLM, we considered several aspects:

- **Cost:** The financial implications of model utilization vary significantly depending on the deployment strategy. For models operated on private infrastructure, the primary cost consideration involves the expenses associated with cloud services. Alternatively, when employing a proprietary model accessible via API, the cost per token becomes a pivotal factor. Opting for the latter, our strategy focused on crafting concise prompts to minimize expenses without compromising the model's effectiveness.

- **Latency:** The response time of models can range widely, influenced by factors such as model size, architectural complexity, and the computational power of the hosting environment. This variance is a critical consideration, especially in scenarios requiring rapid iterative testing and feedback. Although larger, more computationally intensive models may offer superior performance, selecting a model that balances response time and computational demands was essential for our workflow. In our approach, we utilized both, the efficiency of climateBERT to rapidly iterate over training examples and then the large context of a GPT model for the final classification.

- **Performance for Specific Tasks:** The adaptability of modern LLMs to a broad spectrum of tasks is remarkable, often eliminating the need for fine-tuning or complex prompting strategies for general applications. However, specialized tasks may necessitate tailored adjustments or fine-tuning to achieve optimal results. The trade-off between using generalized versus specialized language models for niche domains has been explored in research, such as in medicine[8] and finance[9].

Given these considerations, our choice of the final model was informed by a holistic assessment of primarily task-specific performance, cost, and latency. Despite ClimateBERT's suitability for our initial training needs, we use GPT-3.5 as our final classification model because of its superior performance. We compare performance of the different fine-tuned models using five-fold cross-validation and show the average F1 and accuracy scores across five folds. We use a batch size of 8 and train for 10 epochs, we select the ClimateBERT model with the highest F1 score by conducting a grid search over key hyperparameters, including learning rates (5e-05, 2e-05, 1e-05, 5e-06), epsilons (1e-08, 1e-07), and dropout probabilities (0.1, 0.2, 0.3). The optimal model for which our accuracy and F1 scores are based on has a learning rate of 5e-05, epsilon of 1e-08, and dropout of 0.1. We also benchmark with four additional models, DistilRoBERTa, RoBERTa, DeBERTa, and EnvironmentalBERT[10–13]. For these models, we use the same training parameters as the ClimateBERT model, as they share similar Transformer-based model architectures and exhibit stable performance under comparable tuning configurations. One exception is for the RoBERTa model, which collapsed into a single-class prediction when trained with a learning rate of 5e-05, hence we reduced the learning rate to 5e-06 to allow the model to train more gradually and avoid premature convergence. Across these alternative models, the F1 and accuracy rates are below the fine-tuned GPT-3.5 we use (Supplementary Table 11). For example, the average accuracy for the fine-tuned GPT-3.5 is at 84.09%, which is higher than that of the fine-tuned ClimateBERT's accuracy of 81.98%. In particular, the fine-tuned GPT-3.5 outperforms the other models more in correctly identifying climate solutions sentences in industries with fewer climate solutions, such as in Equity Real Estate Investment Trusts and Household and Personal Products. In summary, by fine-tuning GPT-3.5-turbo-1106 with a targeted training set, we achieve a balance between cost efficiency and performance.

A challenge with utilizing GPT-3.5 is its non-deterministic behavior. Non-deterministic behav-

ior in GPT refers to the variability in its outputs even when given the same input multiple times. This behavior arises from several factors inherent to the design and operation of the model. One key factor is temperature, which controls the randomness of predictions. A higher temperature value (e.g., 1.0) produces more random outputs, while a lower temperature (e.g., 0.1) makes the output more deterministic and focused on high-probability tokens (refer to the words or sub-words that the model predicts are most likely to come next in a given sequence). Therefore, to reduce variability in predictions, we set the temperature hyperparameter to 0.1. Additionally, to examine the potential variability in this non-deterministic behavior, we randomly selected 10,000 sentences from outside the labeled dataset and apply the fine-tuned GPT model five times. The maximum discrepancy observed between any two columns was 14 rows, or 0.14%. Additionally, we attempt to mitigate the concern about non-determinism in the main results by conducting sensitivity analyses to test the robustness of our findings. We introduce random variation to the Climate Solutions Measure across the full sample to resemble the 0.14% non-determinism and re-run our regression analyses. Specifically, we add normally distributed noise with a mean of zero and a standard deviation of 0.14%, 0.28% (2x stretch test), and 1.4% (10x stretch test) to Climate Solutions Measure, and truncate values below zero to maintain interpretable results. The results from the regression analysis in the paper remain robust, providing some comfort regarding the potential impact of the non-determinism concern.

Topic Modeling

The climate solutions measure provides an overall view into companies' engagement with climate solutions. To enrich this analysis, we map each climate solutions sentence to 88 climate solutions topics (listed in Supplementary Table 12). To construct the topics, we provide GPT 3.5 (our fine-tuned version) description of climate solutions listed in Project Drawdown, and ask GPT to assign each climate solutions sentence from 10-K Item 1 to the closest topic.

We start with 81 climate solutions listed in the 2020 Project Drawdown Review. For each topic, Project Drawdown provides a description of around 25 words. We provide this list of descriptions in a prompt for the fine-tuned GPT to classify each climate solutions sentence to the closest topic. Our test set contains 700 sample climate solutions sentences that we label the relevant climate solutions topic, and these 700 sentences are chosen to represent each of the climate solutions topics. Based on these observations, we add the following new topics because we notice that the model has difficulty classifying these:

- Fuel cell: while Project Drawdown does include the use of hydrogen as an energy storage technology, fuel cell is not explicitly listed as a climate solution, which we add as a separate topic to ensure consistent classification.
- Sustainable aviation fuel: while Project Drawdown does include the use of biomass power, sustainable aviation fuel is not explicitly listed as a climate solution, which we add as a separate topic to ensure consistent classification.
- Cogeneration: while Project Drawdown does include the use of high-efficiency heat pumps, cogeneration, also known as Combined Heat and Power (CHP), is not explicitly listed as a climate solution, which we add as a separate topic to ensure consistent classification.
- Renewable energy: some sentences contain multiple renewable energy sources and some sentences only describe renewable energy without specifying the source. Hence, we create a new topic for renewable energy to ensure consistent classification.
- Energy efficiency: some sentences describe other sources of energy efficiency beyond the

specific examples listed in Project Drawdown (such as high-efficiency heat pumps). Hence, we create a new topic for energy efficiency to ensure consistent classification.

- Natural gas: while the use of natural gas is not a climate solution listed in Project Drawdown, and our labeling process does not classify it as a climate solution, some sentences about natural gas are classified as climate solutions sentences when they describe the use of natural gas to lower emissions when replacing coal production. As such, we separately include a topic for natural gas.

- Batteries in electric vehicles: some sentences mention both electric vehicles and the use of batteries, which the model does not classify consistently. Hence, we include this as a separate topic to ensure consistent classification, and then combine this topic with distributed energy storage, which is an existing solution listed in Project Drawdown.

- Carbon capture and storage: while Project Drawdown does not list carbon capture and storage as a solution, it acknowledges the plan to expand solutions in this space. This topic was initially not included in the list of topics provided to GPT, but the model commonly includes this topic in the output, and hence we add this as an additional topic.

After refining the prompt and adding these topics, we achieve an accuracy rate of 88% for the 700-sentence training set using the fine-tuned GPT. We repeat the procedure three times and only 8 sentences disagree. In contrast, when we use the equivalent GPT model without fine-tuning, the model achieves a lower accuracy of 74% and produces 75 disagreements across three repeated runs. One possible reason for this difference is that the fine-tuned model has a stronger domain understanding of climate solutions, yielding more consistent classifications[14]. As such, we use the fine-tuned GPT for the topic modeling task. Our prompt for the topic modeling task is as follows:

Listing 2: GPT finetuning prompt

```
    system_message =   You   are a chatbot trained to classify
       sentences into one of 88 specific climate solution categories.
        Each category has a unique description that you must use to
       identify the most applicable category for each provided
       sentence.

    Follow these steps:
    - Choose the category that best aligns with the sentence, even if
        the fit isn't perfect.
    - If the sentence applies to multiple categories, select the most
        relevant one.
    - Do not create or suggest categories beyond the predefined 88.

    For ambiguous cases, use your best judgment to select the most
       relevant category based on the primary focus of the sentence.

    Categories:
<A list of solutions categories and descriptions provided here, see
   Supplementary Table 12>
Below is a general example to illustrate the expected output:
Example Sentence: "We also manufacture biodiesel at this facility."
Expected Output: "Biomass Power"

    Please return the category name exactly as listed and provide
       your response in this format: [Category Name]
```

We then apply this prompt to all climate solutions sentences from 10-K Item 1. We repeat this process twice, and only 2.43% of sentences result in two different topics. In our final topic classification, we only keep the topics where the two runs agree to ensure the accuracy of classification. The final list of climate solutions topics, definitions, and frequency are detailed in Supplementary Table 12.

Supplementary Table 12: Climate Solutions Topics

Topic	Topic Group	Description	Mean
Nuclear Power	Alternative Fuel	Nuclear power is slow, expensive, risky, and creates radioactive waste, but it has the potential to avoid emissions from fossil fuel electricity.	0.149
Natural gas	Alternative Fuel	Natural gas serves as a transitional fuel in the shift towards a less carbon-intensive energy system. It burns cleaner than coal and oil, producing fewer pollutants and lower levels of CO2 emissions. This makes natural gas an effective bridge fuel for reducing environmental impact while renewable energy capacities are being scaled up.	0.050
Alternative Cement	Alternative Materials	Cement production requires significant energy and decarbonization of limestone. Fly ash, a waste product from burning coal, can replace some of that material and cut emissions.	0.014
Alternative Refrigerants	Alternative Materials	Fluorinated gases are not the only refrigerants available. Alternatives, such as ammonia or captured carbon dioxide, can replace these powerful greenhouse gases over time.	0.011
Bioplastics	Alternative Materials	Most plastics are made from fossil fuels, but bioplastics utilize plants as an alternative source of carbon. They often have lower emissions and sometimes biodegrade. One example is bio-succinic acid.	0.066
Plant-Rich Diets	Alternative Materials	Consumption of meat and dairy, as well as overall calories, often exceeds nutritional recommendations. Paring down and favoring plant-based foods reduces demand, thereby reducing land clearing, fertilizer use, burping cattle, and greenhouse gas emissions. Examples include plant-based protein and dairy-free products.	0.010
Electric Cars	Electrification	Electric motors supplant gasoline or diesel engines, which are polluting and less efficient. EVs always reduce car emissions—dramatically so when powered by renewable electricity.	0.162
Electric Trains	Electrification	Rail electrification enables trains to move beyond dirty dieselburning engines. When powered by renewables, electric trains can provide nearly emissions-free transport.	0.001
High-Efficiency Heat Pumps	Electrification	Heat pumps extract heat from the air and transfer it—from indoors out for cooling, or from outdoors in for heating. With high efficiency, they can dramatically lower building energy use.	0.005
Hybrid Cars	Electrification	A transitional technology, hybrid cars pair an electric motor and battery with an internal combustion engine. The combination improves fuel economy—more miles on a gallon—and lowers emissions.	0.019
Distributed Energy Storage	Energy storage	Standalone batteries and electric vehicles store energy. They can enable 24/7 electricity supply even when the sun isn't shining or the wind isn't blowing.	0.102
Utility-Scale Energy Storage	Energy storage	Large-scale energy storage ensures electricity supply can match demand. It enables the shift to variable renewables and curbs emissions from polluting "peaker" plants.	0.053
Fuel cell	Energy storage	Fuel cells generate electricity through a chemical reaction, typically involving hydrogen and oxygen, without combustion. This clean technology offers high energy efficiency compared to traditional energy sources and emits only water as a byproduct. Fuel cells are used in a range of applications from powering vehicles to providing emergency electricity for buildings and as part of larger renewable energy systems.	0.070
Building Automation Systems	Enhance Efficiency	These systems can control heating, cooling, lighting, and appliances in commercial buildings. They cut emissions by maximizing energy efficiency and minimizing waste.	0.016
Building Retrofitting	Enhance Efficiency	Retrofits address electricity and fuel waste with better insulation and windows, efficient lighting, and advanced heating and cooling systems. Improved efficiency lowers existing buildings' emissions.	0.017
Carpooling	Enhance Efficiency	When people share common origins, destinations, or stops en route, they can ride together. Carpooling uses seats and fuel more efficiently, cutting emissions.	0.000
District Heating	Enhance Efficiency	District systems heat space and water more efficiently. A central plant and pipe network channel hot water to many buildings, with lower emissions than on-site systems.	0.000
Dynamic Glass	Enhance Efficiency	By responding to sunlight and weather, dynamic glass can reduce a building's energy load for heating, cooling, and lighting. More effective windows lower emissions.	0.000

Continued on following page

Supplementary Table 12, continued

Topic	Topic Group	Description	Mean
Efficient Aviation	Enhance Efficiency	Various technologies and operational practices can lower airplane emissions to some degree. They include better engines, wingtips, and light weighting to improve fuel efficiency.	0.004
Efficient Ocean Shipping	Enhance Efficiency	Huge volumes of goods are shipped across oceans. Fuel-saving ship design, onboard technologies, and operational practices can improve efficiency and trim emissions.	0.001
Efficient Trucks	Enhance Efficiency	Fuel-efficiency is critical to reduce road-freight emissions. Existing fleets can be retrofitted, while new trucks can be built to be more efficient or fully electric.	0.002
Farm Irrigation Efficiency	Enhance Efficiency	Pumping and distributing water is energy intensive. Drip and sprinkler irrigation, among other practices and technologies, make farm-water use more precise and efficient.	0.000
Green and Cool Roofs	Enhance Efficiency	Green roofs use soil and vegetation as living insulation. Cool roofs reflect solar energy. Both reduce building energy use for heating and/or cooling.	0.001
High-Performance Glass	Enhance Efficiency	High-performance glass improves window insulation and makes building heating and cooling more efficient. By minimizing unnecessary energy use, it curtails emissions.	0.006
Insulation	Enhance Efficiency	Insulation impedes unwanted airflow in or out of buildings. In new construction or retrofits, it makes heating and cooling more energy efficient, with lower emissions.	0.003
LED Lighting	Enhance Efficiency	LEDs (light emitting diodes) are the most energy-efficient bulbs available. Unlike older technologies, they transfer most of their energy use into light, rather than waste heat.	0.076
Low-Flow Fixtures	Enhance Efficiency	Cleaning, transporting, and heating water requires energy. More efficient fixtures and appliances can reduce home water use significantly, thereby reducing emissions.	0.000
Net Zero Buildings	Enhance Efficiency	Buildings with zero net energy consumption combine maximum efficiency and onsite renewables. They produce as much energy as they use annually, with low or no emissions.	0.006
Nutrient Management	Enhance Efficiency	Overuse of nitrogen fertilizers—a frequent phenomenon in agriculture—creates nitrous oxide. More efficient use can curb these emissions and reduce energy-intensive fertilizer production. Examples include biofertilizers or bioinsecticides.	0.002
Reduced Food Waste	Enhance Efficiency	Roughly a third of the world's food is never eaten, which means land and resources used and greenhouse gases emitted in producing it were unnecessary. Interventions can reduce loss and waste, as food moves from farm to fork, thereby reducing overall demand.	0.000
Refrigerant Management	Enhance Efficiency	Fluorinated gases have a potent greenhouse effect and are widely used as refrigerants. Managing leaks and disposal of these chemicals can avoid emissions in buildings and landfills.	0.004
Smart Thermostats	Enhance Efficiency	Thermostats are mission control for space heating and cooling. Smart thermostats use algorithms and sensors to become more energy efficient over time, lowering emissions.	0.000
Sustainable Intensification for Smallholders	Enhance Efficiency	Sustainable intensification practices can increase smallholder yields, which, in theory, reduce demand to clear additional land. Practices include intercropping, ecosystem-based pest management, and equal resources for women.	0.001
System of Rice Intensification	Enhance Efficiency	SRI is a holistic approach to sustainable rice cultivation. By minimizing water use and alternating wet and dry conditions, it minimizes methane production and emissions.	-
Water Distribution Efficiency	Enhance Efficiency	Pumping water requires enormous amounts of electricity. Addressing leaks in water-distribution networks, especially in cities, can curb water loss, energy use, and emissions.	0.000
Cogeneration	Enhance Efficiency	Cogeneration, also known as Combined Heat and Power (CHP), is an efficient energy solution that simultaneously generates electricity and useful thermal energy from a single fuel source. Examples include microturbines and heat recovery.	0.040
Energy efficiency	Enhance Efficiency	This category covers instances where the term energy efficiency is involved but it does not fit any of the preexisting categories.	0.312
Abandoned Farmland Restoration	Nature	Degraded farmland is often abandoned, but need not be. Restoration can bring these lands back into productivity and sequester carbon in the process.	0.003

Continued on following page

Supplementary Table 12, continued

Topic	Topic Group	Description	Mean
Bamboo Production	Nature	Bamboo rapidly sequesters carbon in biomass and soil and can thrive on degraded lands. Long-lived bamboo products can also store carbon over time.	0.002
Biochar Production	Nature	Biomass slowly baked in the absence of oxygen becomes biochar, retaining most of the feedstock's carbon. It can be buried for sequestration and potentially enrich soil.	0.005
Coastal Wetland Protection	Nature	Mangroves, salt marshes, and seagrasses sequester huge amounts of carbon in plants and soil. Protecting them inhibits degradation and safeguards their carbon sinks.	0.000
Coastal Wetland Restoration	Nature	Agriculture, development, and natural disasters have degraded many coastal wetlands. Restoring mangrove forests, salt marshes, and seagrass beds to health revives carbon sequestration.	0.000
Conservation Agriculture	Nature	Conservation agriculture uses cover crops, crop rotation, and minimal tilling in the production of annual crops. It protects soil, avoids emissions, and sequesters carbon.	0.000
Forest Protection	Nature	In their biomass and soil, forests are powerful carbon storehouses. Protection prevents emissions from deforestation, shields that carbon, and enables ongoing carbon sequestration.	0.001
Grassland Protection	Nature	Grasslands hold large stocks of carbon, largely underground. Protecting them shields their carbon stores and avoids emissions from conversion to agricultural land or development.	0.000
Improved Rice Production	Nature	Flooded rice paddies produce large quantities of methane. Improved production techniques, including alternate wetting and drying, can reduce methane emissions and sequester carbon.	0.000
Indigenous Peoples' Forest Tenure	Nature	Secure land tenure protects Indigenous peoples' rights. With sovereignty, traditional practices can continue—in turn protecting ecosystems and carbon sinks and preventing emissions from deforestation.	-
Managed Grazing	Nature	Managed grazing involves carefully controlling livestock density, and timing and intensity of grazing. Compared with conventional pasture practices, it can improve the health of grassland soils, sequestering carbon.	0.000
Multistrata Agroforestry	Nature	Multistrata agroforestry systems mimic natural forests in structure. Multiple layers of trees and crops achieve high rates of both carbon sequestration and food production.	0.000
Peatland Protection and Rewetting	Nature	Forestry, farming, and fuel-extraction are among the threats to carbon-rich peatlands. Protection and rewetting can reduce emissions from degradation, while supporting peatlands' role as carbon sinks.	0.000
Perennial Staple Crops	Nature	Perennial staple crops provide important foods, such as bananas, avocado, and breadfruit. Compared to annual crops, they have similar yields but higher rates of carbon sequestration.	0.001
Regenerative Annual Cropping	Nature	Building on conservation agriculture with additional practices, regenerative annual cropping can include compost application, green manure, and organic production. It reduces emissions, increases soil organic matter, and sequesters carbon.	0.001
Silvopasture	Nature	An agroforestry practice, silvopasture integrates trees, pasture, and forage into a single system. Incorporating trees improves land health and significantly increases carbon sequestration.	-
Temperate Forest Restoration	Nature	Almost all temperate forests have been altered in some way—timbered, converted to agriculture, disrupted by development. Restoring them sequesters carbon in biomass and soil.	0.000
Tree Intercropping	Nature	Growing trees and annual crops together is a form of agroforestry. Tree intercropping practices vary, but all increase biomass, soil organic matter, and carbon sequestration.	0.000
Tree Plantations (on Degraded Land)	Nature	Degraded lands present potential locations for tree plantations. Managed well, they can restore soil, sequester carbon, and produce wood resources in a more sustainable way.	0.001
Tropical Forest Restoration	Nature	Tropical forests have suffered extensive clearing, fragmentation, degradation, and depletion of biodiversity. Restoring these forests also restores their function as carbon sinks.	0.000
Concentrated Solar Power	Renewable	Concentrated solar power uses sunlight as a heat source. Arrays of mirrors concentrate incoming rays onto a receiver to heat fluid, produce steam, and turn turbines.	0.004

Continued on following page

Supplementary Table 12, continued

Topic	Topic Group	Description	Mean
Distributed Solar Photovoltaics	Renewable	Rooftop solar panels are one example of distributed solar photovoltaic systems. Whether grid-connected or part of stand-alone systems, they offer hyper-local, clean electricity generation.	0.078
Geothermal Power	Renewable	Underground reservoirs of steamy hot water are the fuel for geothermal power. It can be piped to the surface to drive turbines that produce electricity without pollution.	0.024
Grid Flexibility	Renewable	Smarter, more flexible electric grids can cut energy losses during distribution. They are critical to enable renewables, which are more variable than conventional electricity generation. One example is smart grid.	0.014
Microgrids	Renewable	A microgrid is a localized grouping of distributed electricity generation technologies, paired with energy storage or backup generation and tools to manage demand or "load."	0.003
Micro Wind Turbines	Renewable	Micro wind turbines can generate clean electricity in diverse locations, from urban centers to rural areas without access to centralized grids.	0.001
Ocean Power	Renewable	Wave- and tidal-power systems harness natural oceanic flows—among the most powerful and constant dynamics on earth—to generate electricity without pollution.	0.008
Offshore Wind Turbines	Renewable	Winds over sea are more consistent than those over land. Offshore wind turbines tap into that power to generate utility-scale electricity without emissions.	0.006
Onshore Wind Turbines	Renewable	Onshore wind turbines generate electricity at a utility scale, comparable to power plants. They replace fossil fuels with emissions-free electricity.	0.057
Small Hydropower	Renewable	Small hydropower systems capture the energy of free-flowing water, without using a dam. They can replace dirty diesel generators with clean electricity generation. Include hydroelectric power in this category.	0.019
Solar Hot Water	Renewable	Solar hot water taps the sun's radiation, rather than fuel or electricity. By replacing conventional energy sources with a clean alternative, it reduces emissions.	0.002
Utility-Scale Solar Photovoltaics	Renewable	Solar photovoltaics can be used at utility-scale—with hundreds or thousands of panels—to tap the sun's clean, free fuel and replace fossil fuel electricity generation.	0.164
Renewable energy	Renewable	This category covers instances where the term 'renewable' is used without specifying a type, or when multiple types of renewable energy sources, such as solar, wind, and hydro, are mentioned together. If only one type of renewable energy source is mentioned, please use the more specific category.	0.412
Biogas for Cooking	Use Waste	Anaerobic digesters process backyard or farmyard organic waste into biogas and digestate fertilizer. Biogas stoves can reduce emissions when replacing biomass or kerosene for cooking.	0.021
Biomass Power	Use Waste	Biomass feedstock can replace fossil fuels for generating heat and electricity. Only perennial biomass is advisable, offering a "bridge" solution to clean, renewable production. Examples include biodiesel and ethanol.	0.244
Clean Cooking	Use Waste	Improved clean cookstoves can address the pollution from burning wood or biomass in traditional stoves. Using various technologies, they reduce emissions and protect human health.	0.000
Composting	Use Waste	Composting can range from backyard bins to industrial-scale operations. Regardless, it converts organic waste into soil carbon, averting landfill methane emissions in the process.	0.001
Landfill Methane Capture	Use Waste	Landfills generate methane as organic waste decomposes. Rather than getting released as emissions, that methane can be captured and used to produce electricity.	0.006
Methane Digesters	Use Waste	Industrial-scale anaerobic digesters control decomposition of organic waste and convert methane emissions into biogas, an alternative fuel, and digestate, a nutrient-rich fertilizer.	0.001
Perennial Biomass Production	Use Waste	Bioenergy relies on biomass—often annual crops such as corn. Perennial plants (e.g., switchgrass, silvergrass, willow, eucalyptus) are a more sustainable source and sequester modest amounts of soil carbon.	0.004
Recycled Paper	Use Waste	Recycled paper takes a circular journey, rather than a linear flow from logging to landfill. Reprocessing used paper curtails extraction of virgin feedstock and lowers emissions.	0.015
Recycling	Use Waste	To produce new products from recovered materials requires fewer raw resources and less energy. That's how recycling household, commercial, and industrial waste can cut emissions.	0.042

Continued on following page

Supplementary Table 12, continued

Topic	Topic Group	Description	Mean
Waste to Energy	Use Waste	Waste-to-energy processes (incineration, gasification, pyrolysis) combust waste and convert it to heat and/or electricity. Emissions reductions can come with health and environmental risks, however.	0.020
Sustainable Aviation Fuel	Use Waste	Sustainable Aviation Fuel (SAF) is a type of biofuel used to power aircraft that is designed to be more environmentally friendly than conventional jet fuel. SAF is derived from sustainable resources such as cooking oil, plant waste, and other biomass.	0.005
Bicycle Infrastructure	Other	Bicycles offer an alternative to cars and fossil fuel transport, especially in cities. Infrastructure is essential for supporting safe and abundant bicycle use, thereby curbing emissions.	0.000
Carbon Capture and Storage	Other	Carbon Capture and Storage (CCS) is a technology designed to capture and store carbon dioxide (CO2) emissions from industrial processes and power generation. The captured CO2 is then transported and stored underground in geological formations to prevent its release into the atmosphere, thereby reducing greenhouse gas emissions.	0.018
Electric Bicycles	Other	Small battery-powered motors give electric bicycles a boost. It makes them a more compelling alternative to more polluting forms of motorized transport, namely cars.	0.003
High-Speed Rail	Other	High-speed rail offers an alternative to trips otherwise made by car or airplane. It requires special, designated tracks, but can dramatically curtail emissions.	0.000
Public Transit	Other	Streetcars, buses, and subways offer alternative, efficient modes of transport. Public transit can keep car use to a minimum and avert greenhouse gases.	0.001
Telepresence	Other	Telepresence integrates high-performance visual, audio, and network technologies, so people can interact across geographies. It cuts down on travel—especially flying—and its emissions.	0.000
Walkable Cities	Other	Walkable cities use planning, design, and density to maximize walking and minimize driving, especially for commuting. Emissions decrease as pedestrians take the place of cars.	0.000

This table provides the descriptions of climate solutions topics, and the percent of the topic out of all 10-K item 1 sentences for the average firm-year observation.

Supplementary Note 2: Climate Solutions Labeling

To train our GPT climate solutions model, we label 3,508 sentences as either climate solution sentences or not. For our annotation procedure, we implement the following general rules referencing Webersinke et al., 2022[6]. The annotators have to determine whether a sentence is related to climate solutions. Annotators are asked to apply common sense, e.g., when a given sentence might not provide all the context, but the context might seem obvious. Moreover, annotators are informed that each annotation should be a 0-1 decision. Hence, if an annotator is 70% certain, it is rounded up to 100%. Two researchers annotate the same tasks to obtain some measure of dispersion. In case of a close verdict or a tie between the annotators, the authors of this paper discuss the sentence in depth before reaching an agreement. Out of 3,508 sentences, annotators agreed on 2,905, while the remaining sentences had disagreements. To assess the degree of annotator agreement, we calculate Cohen's Kappa, which is 0.6653 with a 95% confidence interval of 0.64 to 0.6907. This indicates a substantial level of agreement in the labeling process.

We define climate solutions as products and services that develop or deploy new technologies in a transition to a low-carbon economy. As a general rule, we determine that just discussing climate change or the environment is not sufficient, the sentence should mention specific climate solutions, such as renewable energy, electrification of transportation and processes, battery technology, new agricultural practices, or plant-based protein alternatives to meat. When in doubt, we refer to the list of climate solutions technologies listed in Project Drawdown. Below, we provide some examples.

**Sentence**	**Label**	**Reason**
Our industry experience, the performance of our transit buses, and compelling total cost of ownership has helped make us the leader in the U.S. electric transit bus market.	1	The firm is creating electric transit bus, which, as an electric vehicle, is a climate solution.
We believe we have a responsibility and opportunity to play a role in the global economic transition to net zero emissions.	0	This is a generic statement without referencing specific products or investments, as compared to the previous sentence.
Our expanding corporate offices in Los Angeles, California are being designed and developed to qualify for LEED certification.	0	This is about their current operations, and not a product they are developing.
Many of our products meet the requirements for the awarding of LEED credits, and we are continuing to develop new products, systems and services to address market demand for products that enable construction of buildings that require fewer natural resources to build, operate and maintain.	1	This is similar to the last sentence in mentioning the LEED certification, but is used with respect to a product, and therefore qualifies.
The first class of QFs includes energy producers that generate power using renewable energy sources such as wind, solar, geothermal, hydro, biomass or waste fuels.	0	This reads as part of a regulation for Qualifying Facilities, and not a product or any indication of a company's actions.

Special cases

From the labeling effort, a few common themes come up frequently, which merit some discussion. We hope that these examples make clear some of the choices that are made during the discussions when disagreements arise.

Ownership

Ownership (even partial) in a climate solution, such as through acquisitions or purchasing of renewable energy facilities, are considered qualifying sentences.

Sentence	Label	Reason
We increased our original 32% ownership and obtained control of the California renewable fuels facility after.	1	In this case, the company is investing more into renewable energy generation, which is a climate solution.
We are currently evaluating the use of renewable energy to complement our existing supply.	0	This expands on a future plan to utilize solar but not to produce solar or a climate solution product.

Competition and Industry Trends

Sentences that imply a firm belongs to the same market that relates to climate solutions offer a chance to attribute the properties of that shared market to the firm by extension.

Sentence	Label	Reason
Primary fleet EV competitors include Smith Electric, Azure Dynamics, Enova, and EnVision Motor Company.	1	This sentence implies that the company in question also manufactures electric vehicles (like its competitors).
A number of important factors are contributing to the trend of growth in the electrification of the light duty commercial vehicle segment.	0	The sentence does reference a qualifying industry segment, but fails to attribute the segment to the company or product.

Carbon Credits and Certifications

Sentences that imply products are sustainable or a low-carbon alternative, either by generating carbon credits, or some other type of industry-level certification are acceptable ways to infer a climate solution product.

Sentence	Label	Reason
In August 2011, we commenced the registration process for the first two landfill projects... to be submitted to the Executive Board of the Clean Development Mechanism for our projects to be registered and receive carbon credits.	1	We can assume that given the facility is generating carbon credits, that it is a climate solutions project.
Consistent with our environmental focus, our electric dispensers are Energy Star rated, and, we believe, utilize less energy than competing water dispensers without this industry rating.	1	This company's product has the energy star rating, and there is a claim of reducing energy utilisation, and thus classified as a climate solution.

Extended supply chain and product intent

There are cases where sentences imply a climate solution by referencing another activity in the supply chain that has a direct or indirect connection to a climate solution. When indirect connections exist, we use 'intent' in the sentence to help disambiguate between climate solutions and not climate solutions. We also use intent when there are ambiguous climate solutions present, such as in the efficiency space.

Sentence	Label	Reason
Projects under exploration may have a discovery well or do not have a geothermal resource discovery occurrence yet, but have significant thermal and other physical evidence that warrants the expenditure of capital in search of the discovery of a geothermal resource.	1	This company is investing in upstream operations for geothermal energy.
Our DTO is primarily used as an emulsifier for metalworking fluids and lubricants, in which our product offers improved performance attributes and, in many cases, offers a more sustainable alternative by replacing less environmentally friendly hydrocarbon-based chemicals.	1	The product intent is reflected in the statement about reducing the utilisation of hydrocarbon-based chemicals.
Further, ICEs virtual and augmented reality technology, including applications for phones, tablets and PCs, also allows project stakeholders in different physical locations to meet in real time to visualize, move about, interact.	0	In several contexts, digital solutions like these are considered 'enabling a low carbon transition'. For us in this case, the sentence itself contains no climate solution intent, and so is not a qualifying sentence.
Taken as a portfolio, our natural gas power plants are among the most efficient in converting natural gas to power and emit far fewer pollutants than most typical utility fleets.	0	In general, we do not consider oil and gas efficiency as a climate solution, and no intent for climate solution development is found in the sentence.

Specific Technologies or Terminologies

There are sentences that describe specific technologies or terminologies that may relate to lowering emissions. We refer to the Drawdown Project when in doubt, and have an internal discussion about each of them, and below provide a few specific examples. Additionally, for specific proper nouns that have a direct tie to a climate solution, we err on the side of only qualifying sentences that make that tie clear in their own business descriptions. Tesla's 'Gigafactory' is an example of a proper noun classified as a climate solution.

**Sentence**	**Label**	**Reason**
In our Interactive Distributed Generation business, we are very focused on the needs of utilities, and partner with utilities to develop, market and manage distributed generation systems for their customers.	1	While distributed generation refers to on-site energy generation, since most of these are renewable energy, we consider this a climate solutions sentence.
The Ambient smart grid platform, known as Ambient Smart Grid, facilitates a two-way, real-time communications network to serve the last mile backhaul.	1	Smart grid is classified as a climate solution in Project Drawdown.
The remaining generation (42%) came from our interest in the nuclear Plants Vogtle and Hatch which would likely not be impacted by any climate change regulation or legislation aimed at reducing greenhouse gas emissions.	1	We consider nuclear power as a climate solution as it is listed in Project Drawdown.

Supplementary References

[1] Ewens, M., Peters, R. H. & Wang, S. Measuring intangible capital with market prices. *Management Science* **71**, 1–953 (2025).

[2] Iqbal, A., Rajgopal, S., Srivastava, A. & Zhao, R. A better estimate of internally generated intangible capital. *Management Science* **71**, 731–752 (2025).

[3] Glaeser, S. The effects of proprietary information on corporate disclosure and transparency: Evidence from trade secrets. *Journal of Accounting and Economics* **66**, 163–193 (2018).

[4] Sautner, Z., Van Lent, L., Vilkov, G. & Zhang, R. Firm-level climate change exposure. *The Journal of Finance* **78**, 1449–1498 (2023).

[5] Hoberg, G. & Phillips, G. Text-based network industries and endogenous product differentiation. *Journal of Political Economy* **124**, 1423–1465 (2016).

[6] Webersinke, N., Kraus, M., Bingler, J. A. & Leippold, M. ClimateBert: A pretrained language model for climate-related text. *arXiv* (2022). https://arxiv.org/abs/2110.12010.

[7] Stammbach, D., Webersinke, N., Bingler, J. A., Kraus, M. & Leippold, M. Environmental claim detection. *arXiv* (2023). https://arxiv.org/abs/2209.00507.

[8] Nori, H. *et al.* Can generalist foundation models outcompete special-purpose tuning? Case study in medicine. *arXiv* (2023). https://arxiv.org/abs/2311.16452.

[9] Li, X. *et al.* Are ChatGPT and GPT-4 general-purpose solvers for financial text analytics? A study on several typical tasks. *arXiv* (2023). https://arxiv.org/abs/2305.05862.

[10] Sanh, V., Debut, L., Chaumond, J. & Wolf, T. DistilBERT, a distilled version of BERT: smaller, faster, cheaper and lighter. *arXiv* (2019). https://arxiv.org/abs/1910.01108.

[11] Liu, Y. *et al.* RoBERTa: A robustly optimized BERT pretraining approach. *arXiv* (2019). https://arxiv.org/abs/1907.11692.

[12] He, P., Liu, X., Gao, J. & Chen, W. DeBERTa: Decoding-enhanced BERT with disentangled attention. *arXiv* (2020). https://doi.org/10.48550/arXiv.2006.03654.

[13] Schimanski, T. *et al.* Bridging the gap in ESG measurement: Using NLP to quantify environmental, social, and governance communication. *Finance Research Letters* **61**, 104979 (2024).

[14] Ouyang, L. *et al.* Training language models to follow instructions with human feedback. *Proceedings of the 36th International Conference on Neural Information Processing Systems* 27730–27744 (2022).
